# Factors Associated with Influenza Vaccination Among Urban Community-Dwelling Chinese Elderly: Results from a Multicity Cross-Sectional Study

**DOI:** 10.3390/vaccines13111171

**Published:** 2025-11-18

**Authors:** Jiayue Guo, Xitong Jiao, Shuai Yuan, Lili You

**Affiliations:** 1 School of Health Policy and Management, Chinese Academy of Medical Sciences and Peking Union Medical College, Beijing 100080, China; s2023026017@pumc.edu.cn (J.G.); s2023026018@pumc.edu.cn (X.J.); 2 Department of Health Policy and Management, Bloomberg School of Public Health, Johns Hopkins University, Baltimore, MD 21205, USA; shuai.yuan@jhu.edu; 3 Institute for Hospital Management, Tsinghua University, Shenzhen 518000, China

**Keywords:** China, elderly, factors, influenza, vaccination, co-occurrence network, cluster analysis

## Abstract

**Background:** Influenza vaccination reduces morbidity and mortality in older adults. This study identifies characteristics and reasons for vaccination uptake among the elderly to inform strategies to improve coverage. **Methods:** We conducted a cross-sectional survey in December 2024 among community-dwelling adults aged ≥ 60 years across six Chinese cities. Data collected included socio-demographic and health characteristics, influenza vaccine awareness and uptake, reasons for vaccination or non-vaccination, and intentions for future vaccination. Univariate and multivariable logistic regression were used to identify factors associated with vaccination. To explore motivation patterns, co-occurrence networks of vaccination reasons were constructed, and k-medoids clustering was applied. **Results**: Among 13,363 adults aged ≥ 60 years, influenza vaccination coverage was 34.0%. Higher education and income, being married, having health insurance, poor self-care ability, and chronic obstructive pulmonary disease were independently associated with vaccination. Vaccinated individuals reported more positive attitudes and were mainly motivated by family and doctor recommendations as well as perceived vaccine effectiveness, with four motivation profiles discovered: social recommendation, comprehensive confidence, clinician-guided, and self-reliant confidence. Among unvaccinated participants, the main reasons for non-vaccination were mild influenza symptoms and the influence of family and friends, forming four motivation profiles: safety concern, low-perceived risk, social influence, and perceived ineffectiveness. **Conclusions**: Influenza vaccination among older Chinese adults remains suboptimal. Tailored interventions leveraging healthcare provider endorsement, family and social support, and policy-driven strategies such as free or subsidized vaccination are needed, particularly for high-risk populations.

## 1. Introduction

Influenza is a highly contagious yet preventable viral respiratory illness that poses a substantial global health burden, causing considerable annual morbidity and mortality worldwide [1]. Current data indicates that around one billion people catch seasonal influenza annually worldwide, with 3–5 million developing severe complications that lead to 290,000–650,000 respiratory fatalities attributed to the virus [2]. Hospital admissions and deaths predominantly affect seniors aged 60 and above, who face greater risks due to weakened immunity associated with aging and higher rates of chronic health conditions [3]. A China-based study covering the 2010–2011 to 2014–2015 influenza seasons estimated an average of 88,100 excess respiratory deaths annually, with older adults accounting for 80% of these fatalities [4].

Influenza vaccination is a cost-effective strategy for reducing hospitalization and mortality rates among adults aged 65 and above [5]. Routine immunization also decreases outpatient visits for influenza-like symptoms and reduces expenditure on both prescription and over-the-counter medications. Therefore, promoting vaccination among older adults with chronic conditions represents a vital public health priority. Although both the World Health Organization (WHO) and China’s Technical Guidelines for Influenza Vaccination recommend prioritizing adults aged 60 and older for influenza immunization [6], vaccination coverage among Chinese older adults remains suboptimal at 10–30%, falling substantially below recommended levels [7,8,9,10]. In China, older adults (≥60 years) are primarily recommended to receive inactivated influenza vaccines (IIVs), including trivalent (IIV3). For older adults with weakened immune function, high-dose (IIV3-HD) or adjuvanted inactivated vaccines (MF59-adjuvanted IIV3-Adj) may be considered to enhance immunogenicity.

Many countries have successfully incorporated annual influenza vaccination programs into their national immunization plans [11,12]. However, it is noteworthy that in China, the influenza vaccine has not yet been included in the national immunization program and remains self-paid, with costs ranging from 60 to 80 RMB (roughly 9–15 USD). Only a few major cities offer local government-subsidized vaccination initiatives [13,14,15,16]. This situation significantly limits vaccine accessibility, particularly among elderly populations with limited awareness of influenza and constrained financial resources.

This study aims to characterize the socio-demographic profile of older adults receiving influenza vaccination in China and to analyze factors associated with vaccination uptake or non-vaccination, thereby providing evidence for optimizing future influenza vaccination strategies.

## 2. Materials and Methods

### 2.1. Study Design and Participants

In December 2024, we conducted a cross-sectional study across six Chinese cities—Beijing (North), Hangzhou (East), Qingdao (East), Shenzhen (South), Chongqing (Southwest), and Chengdu (Southwest)—selected to ensure broad geographical representation. In each city, five to eight Community Health Service Centers (CHSCs), responsible for providing vaccination services to urban residents, were randomly selected from a comprehensive local list using simple random sampling. The required sample size was estimated using the formula for a single population proportion, assuming an expected vaccination rate of 30%, a 95% confidence level, and a 3% margin of error. After accounting for a design effect of 2 and a 10% non-response rate, at least 2000 participants were required per city.

Each CHSC invited 300 eligible individuals. Elderly residents aged ≥ 60 years attending CHSCs for free physical examinations or vaccinations were consecutively approached in waiting areas and invited to participate. Trained interviewers administered all questionnaires face-to-face using a digital system configured to require completion of all items before submission. Participants provided digital informed consent on the first page of the questionnaire. Each completed questionnaire was assigned a unique reference number and recorded in the system. In total, 13,754 individuals completed the questionnaire.

**Figure 1:**
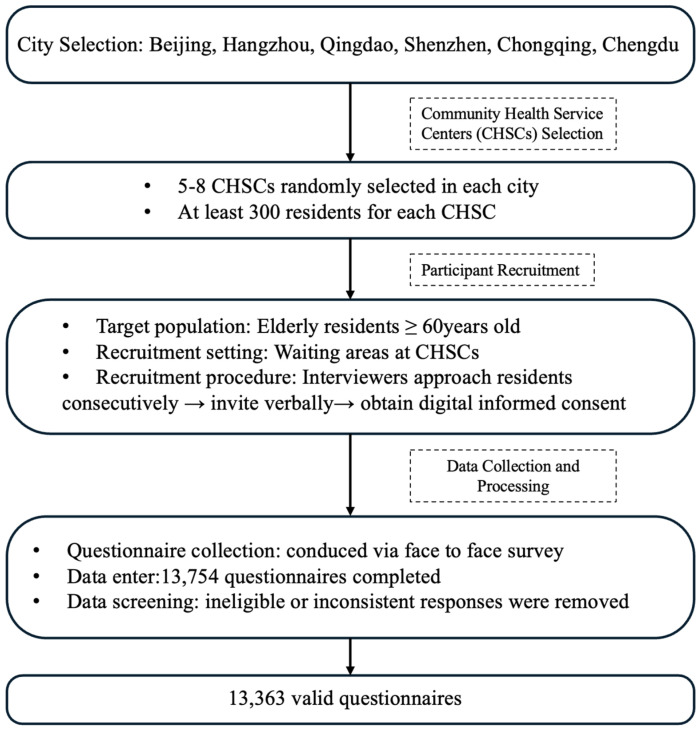
Flowchart of study design and participant recruitment.

During the data processing phase, we applied strict inclusion criteria and logical consistency checks to screen the collected questionnaires. Invalid samples—such as those from respondents who did not meet the age threshold or provided contradictory responses—were removed to ensure the validity of the dataset. A total of 13,363 valid questionnaires were ultimately included, resulting in a valid response rate of 97.2%. The flow of study design and participant recruitment can be seen in Figure 1.

### 2.2. Measures

The questionnaire comprised five sections: (i) socioeconomic and demographic variables; (ii) health status variables; (iii) awareness and attitudes toward influenza vaccination; (iv) reasons for accepting or declining vaccination; (v) intention for revaccination. Details of the questionnaire are provided in Supplementary Material (File S1).

The outcome variable was prior influenza vaccination, assessed by the question, “Have you received an influenza vaccine in the past 12 months?” Responses were binary, with the response categories as “Yes” or “No”. Self-reported vaccination has been shown to be a reliable method for evaluating previous vaccination behavior among older adults [17].

Socio-demographic variables included region, gender, age, education, personal income, marital status, and health insurance coverage. Health status variables covered self-care ability, self-rated health status, number and type of chronic diseases, and self-reported indications for immunization. Attitudes were evaluated using 5-point Likert scales. Vaccinated respondents rated the perceived benefit of the vaccine from “very beneficial” to “not beneficial at all,” while unvaccinated respondents rated their perceived need for vaccination from “very necessary” to “Not necessary at all.” Multi-select questions were used to further explore specific reasons for vaccination or non-vaccination.

### 2.3. Statistically Analysis

Descriptive analyses were conducted to provide an overview of socio-demographic and health-related variables. Influenza awareness and vaccination rates were calculated across different socio-demographic and health status subgroups. Categorical variables were compared using the χ^2^ test, while differences in attitudes between vaccinated and unvaccinated participants were assessed using *t*-tests.

To identify independent factors associated with influenza vaccination, univariate logistic regression analyses were performed, with socio-demographic and health-related characteristics as independent variables. Variables that showed statistical significance or marginal significance (*p* < 0.25, according to the Hosmer and Lemeshow criterion) in the univariate analysis were included in a multivariate logistic regression model using the Enter method. Results were presented as adjusted odds ratios (aORs) with 95% confidence intervals (CIs), and a reference category was specified for each variable. An aOR > 1 indicated a factor that facilitated vaccination, while an aOR < 1 indicated an inhibiting factor. Before performing multivariable logistic regression, multicollinearity among independent variables was assessed using the variance inflation factor (VIF), with a threshold of 10 indicating potential collinearity.

To explore co-occurrence patterns among vaccination reasons, separate networks were constructed for vaccinated and unvaccinated participants. Each network was represented as a binary matrix, with rows corresponding to participants and columns to specific reasons (coded as 1 if selected, 0 otherwise). Node-level centrality measures—including degree, betweenness, closeness, and eigenvector centrality—were calculated. Networks were visualized to display relationships among reasons and identify core factors.

To further analyze vaccination motivation patterns, reasons for both vaccinated and unvaccinated participants were examined using k-medoids clustering via the CLARA (Clustering Large Applications) algorithm. Jaccard distance was used to measure similarity between participants’ responses. To identify the most appropriate number of clusters, models specifying two to five clusters (k = 2–5) were examined and compared. Based on statistical considerations and interpretability, the number of clusters was pre-specified as four (k = 4). Each participant was assigned to a single cluster, representing a group with similar patterns of reasons for accepting or declining vaccination. Cluster-specific proportions of reason endorsement were then calculated and visualized in clustered heatmaps to identify distinct motivational profiles or reasons for non-vaccination.

All statistical analyses were conducted using R version 4.4 (R Foundation for Statistical Computing, Vienna, Austria) and IBM SPSS Statistics version 28 (IBM Corp., Armonk, NY, USA). Two-sided *p*-values < 0.05 were considered statistically significant.

## 3. Results

### 3.1. Subject Characteristics

The mean age of respondents was 69.53 years, with 45.3% male and 54.7% female. Participants were distributed across Chongqing (18.9%), Shenzhen (16.9%), Hangzhou (16.5%), Beijing (16.3%), Qingdao (16.0%), and Chengdu (15.4%). Overall, 66.6% of participants reported at least one chronic condition, with hypertension, diabetes, hyperlipidemia, and stroke being the most common comorbidities (see Table 1 for further sample characteristic). Influenza vaccine awareness was significantly associated with region, age, marital status, education level, income, self-care ability, self-rated health status, and the presence of hypertension or hyperlipidemia. In contrast, gender and certain chronic conditions—including diabetes, stroke, and chronic obstructive pulmonary disease (COPD)—were not significantly associated with vaccine awareness (Table 1).

### 3.2. Vaccination Uptake

Overall, 34.0% of respondents reported having received an influenza vaccine within the past year. Among vaccinated individuals, 75.8% expressed willingness to be revaccinated the following year, whereas only 9.8% of unvaccinated participants intended to receive the vaccine.

Table 2 presents influenza vaccination rates stratified by socio-demographic and health-related characteristics. Among respondents aware of the influenza vaccine, 40.3% were vaccinated, compared with 11.1% among those unaware. Vaccination rates varied significantly across regions (*p* = 0.018), with the highest rate observed in Hangzhou (45.9%), followed by Shenzhen (34.8%), Beijing (32.5%), Chongqing (31.7%), Chengdu (31.3%), and Qingdao (28.0%). No significant gender differences were observed (*p* = 1.000), although males had a slightly higher vaccination rate than females (34.9% vs. 33.4%).

Vaccination rates by age group were 34.2% for 60–64 years, 34.5% for 65–69 years, 33.1% for 70–74 years, 35.2% for 75–79 years, 32.0% for 80–84 years, and 42.3% for those aged ≥85 years, with no statistically significant differences between groups (*p* = 1.000). Rates increased with education level (*p* = 0.018), with the lowest rate among participants with primary education or less (28.0%), and higher rates among those with medium-low (34.6%), medium-high (36.3%), and high education levels (39.6%). Influenza vaccination rates were also positively associated with personal income (*p* = 0.018).

Marital status was significantly associated with vaccination (*p* = 0.018), with married or cohabiting adults showing a higher rate (34.7%) compared with single or widowed participants (29.9%). Vaccination rates were higher among holders of urban employee basic medical insurance (36.0%) and urban-rural resident basic medical insurance (35.1%) than among those without insurance.

Participants with chronic conditions generally had higher vaccination rates. Those with hypertension, diabetes, hyperlipidemia and COPD had rates of 33.8%, 35.4%, 36.4% and 38.9%, respectively. Vaccination rates also increased with the number of chronic conditions: 33.7% among those with one condition, 34.7% with two, and 37.9% with three or more.

Participants reporting better self-care ability had a slightly lower vaccination rate. Vaccination also varied by self-rated health status, with rates of 35.1%, 32.9%, and 33.1% among participants reporting “good,” “fair,” and “poor” health, respectively. Consistent with expectations, older adults meeting criteria for immunization demonstrated significantly higher vaccination rates.

### 3.3. Determinants of Vaccination

No significant multicollinearity was detected. Regarding the socio-demographic factors affecting influenza vaccination among Chinese adults aged 60 years or older, individuals with medium-low education had 18.1% higher odds of vaccination (95% CI: 1.062–1.313, *p* = 0.002), those with medium-high education had 19.3% higher odds (95% CI: 1.060–1.344, *p* = 0.004), and those with high education had 38.2% higher odds (95% CI: 1.196–1.597, *p* < 0.001), compared to those with low education. Higher monthly personal income was also associated with increased vaccination: compared with the ≤2500 CNY group, individuals with an income of 2501–5000 CNY had 58.9% higher odds (95% CI: 1.432–1.764, *p* < 0.001), those with 5001–7500 CNY had 58.3% higher odds (95% CI: 1.383–1.813, *p* < 0.001), and those earning >7500 CNY had 46.8% higher odds (95% CI: 1.236–1.743, *p* < 0.001). Married/cohabiting individuals showed 1.15 times higher vaccination odds than single/widowed persons (95% CI: 1.026–1.296, *p* = 0.016). Regarding health insurance, the OR for vaccination was 1.27 times higher (95% CI: 1.111–1.452, *p* < 0.001) for those with Urban-Rural Resident Basic Medical Insurance, while Urban Employee Basic Medical Insurance showed no significant association with vaccination rate (95% CI: 0.983–1.287, *p* = 0.088). No significant associations were observed between influenza vaccination rates and either gender or age after multivariate adjustment (Table 3).

Table 4 shows the health status factors that affect influenza vaccination among Chinese adults aged 60 years older. After multivariate adjustment, individuals with poor self-care ability had 15.4% higher vaccination odds compared to those with self-care ability (95% CI: 1.041–1.280, *p* = 0.006). Regarding self-assessed health status, those reporting good health showed 21.8% higher vaccination odds than those with poor health (95% CI: 1.067–1.391, *p* = 0.003). For chronic conditions, the OR was 1.13 times higher (CI: 1.026–1.251, *p* = 0.014) in the presence of hyperlipidemia and 1.294 times higher (95% CI: 1.101–1.521, *p* = 0.002) in the presence of respiratory diseases or COPD. However, no significant associations were found for hypertension, diabetes, and cerebrovascular disease or stroke after multivariate adjustment.

### 3.4. Vaccination Motivations and Reasons

Vaccinated participants reported a significantly higher mean attitude score (mean = 4.03) compared with unvaccinated participants (mean = 2.61; *p* < 0.001), indicating more positive perceptions of vaccine benefits among those who had been vaccinated. See details in Supplementary Material (File S2).

**Figure 2:**
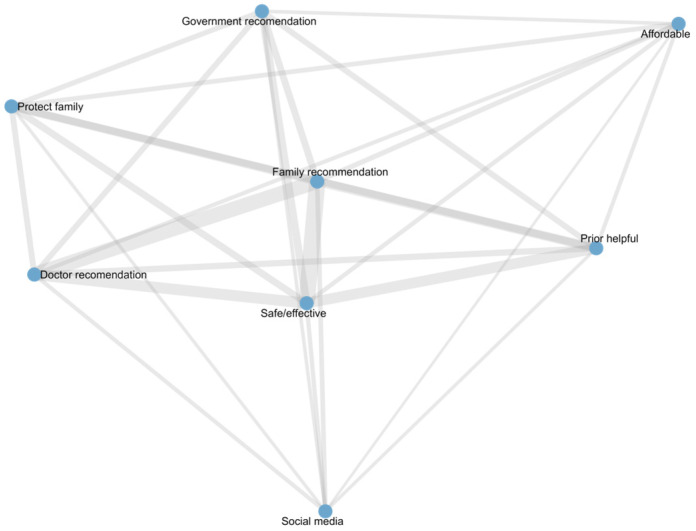
Network of vaccination reasons among vaccinated participants.

Table 5 presents the node-level centrality measures of vaccination reasons in the co-occurrence network constructed from vaccinated participant responses. All reasons had a degree of 7, indicating frequent co-occurrence with other reasons. “Family recommendation”, “Safe/effective”, and “Doctor recommendation” exhibited the highest eigenvector centrality, suggesting they are the core drivers of vaccination decisions. “Social media” showed a high betweenness centrality but lower eigenvector centrality, indicating its role as a bridge connecting different reasons rather than as a core factor. Other reasons, including “Government recommendation”, “Protect family”, and “Affordable”, were moderately connected, contributing to overall network structure but with less central influence. The structure and relationships among these reasons are visualized in the co-occurrence network diagram (Figure 2).

**Figure 3:**
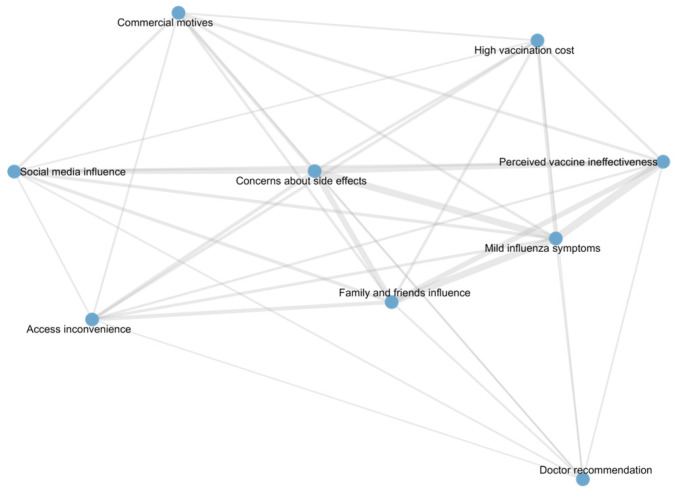
Network of reasons for non-vaccination among unvaccinated participants.

Table 6 shows node-level centrality measures of non-vaccination reasons in the co-occurrence network among unvaccinated participants. All reasons had a degree of 8, indicating frequent co-selection. Core reasons included “Mild influenza symptoms”, “Concerns about side effects”, “Perceived vaccine ineffectiveness”, and “Family and friends influence”, reflecting the main barriers to vaccination. “Doctor recommendation” had the highest betweenness centrality but lower eigenvector centrality, acting as a bridge connecting different combinations of reasons. Other reasons, such as “Social media influence”, “Commercial motives”, “Access inconvenience”, and “High vaccination cost”, were moderately connected, contributing to the network structure but with less central influence. The detailed structure and relationships of these vaccination reasons are illustrated in the co-occurrence network diagram (Figure 3).

**Figure 4:**
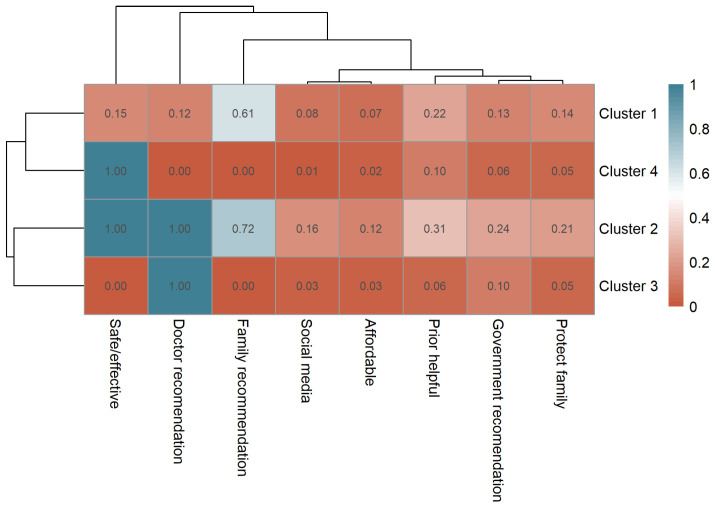
Clustered heatmap of vaccination motivations among vaccinated participants showing four distinct motivation profiles.

Among vaccinated participants, the most frequently selected reason for vaccination was “safe and effective” (n = 2222, 48.8%), followed by “family recommendation” (n = 1617, 35.5%) and “doctor recommendation” (n = 1125, 24.7%). Other reported reasons included “prior helpful experience” (n = 790, 17.4%), “government recommendation” (n = 522, 11.5%), “protecting family” (n = 484, 10.6%), “social media” (n = 282, 6.2%), and “affordable” (n = 241, 5.3%). Vaccination reasons were further analyzed using k-medoids clustering based on Jaccard distance, identifying four clusters (k = 4), each representing a distinct motivational profile (Supplementary Material File S3). The distribution of reasons across clusters is visualized in a clustered heatmap (Figure 4).

Cluster 1 (Family & Social Recommendation Group, n = 2199) represented individuals primarily influenced by family recommendations (mean = 0.61), indicating reliance on social trust rather than professional guidance. Cluster 2 (Comprehensive Confidence Group, n = 393) exhibited the highest endorsement across almost all motivations, including perceived vaccine safety (1.00) and doctor recommendation (1.00), reflecting multi-faceted confidence. Cluster 3 (Clinician-Guided Group, n = 462) was predominantly driven by medical advice (doctor recommendation = 1.00), with minimal influence from other sources. Cluster 4 (Self-Reliant Confidence Group, n = 1496) was characterized by autonomous belief in vaccine effectiveness, reflecting rational self-decision.

**Figure 5:**
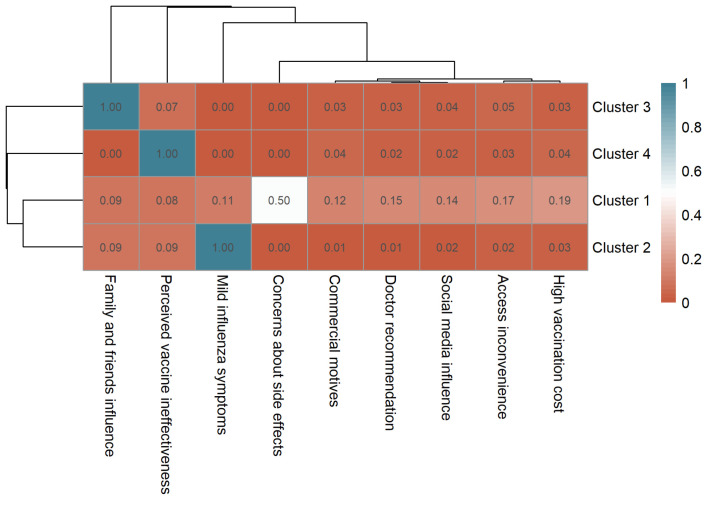
Clustered heatmap of non-vaccination motivations among unvaccinated participants showing four distinct motivation profiles.

Among unvaccinated participants, the most frequently selected reason for not receiving the vaccine was “mild influenza symptoms” (n = 3544, 40.2%), followed by “family and friends influence” (n = 2079, 23.6%), “concerns about side effects” (n = 1642, 18.6%), and “perceived vaccine ineffectiveness” (n = 1526, 17.3%). Other reported reasons included “high vaccination cost” (n = 811, 9.2%), “access inconvenience” (n = 731, 8.3%), “social media influence” (n = 590, 6.7%), “doctor recommendation” (n = 573, 6.5%), and “commercial motives” (n = 520, 5.9%). Clustering analysis similarly identified four distinct participant groups (Supplementary Material, File S4), visualized in a clustered heatmap (Figure 5):

Cluster 1 (Safety Concern Group, n = 3258) showed a high frequency for concerns about side effects (mean = 0.50), representing participants mainly deterred by safety-related issues. Cluster 2 (Low-Perceived Risk Group, n = 3189) was characterized by mild influenza symptoms (mean = 1.00), suggesting participants who did not perceive influenza as a serious illness. Cluster 3 (Social Influence Group, n = 1518) had the highest frequency for family and friends influence (mean = 1.00), indicating decisions primarily shaped by social opinions or discouragement from peers. Cluster 4 (Perceived Ineffectiveness Group, n = 848) reported perceived vaccine ineffectiveness as the main reason for not receiving vaccination.

## 4. Discussion

This multicity cross-sectional study investigated influenza vaccination uptake in the past year among community-dwelling older adults in urban China, shedding light on the diverse factors that drive or hinder their immunization decisions.

The research findings indicate that influenza vaccination coverage among urban Chinese adults aged 60 and above remains suboptimal (28.0–45.9%), falling substantially below the World Health Organization’s 75% target and lower than vaccination rates observed in developed countries [18,19]. Previous research has also shown generally low vaccination coverage among older populations in China [15]. A systematic review reported an average vaccination rate of 51.29% among adults aged 60 and above, with Belgium ranking highest at 72.73%, followed by Hong Kong SAR, China, at 54% [20]. These findings underscore the urgent need to identify factors associated with vaccination behavior and implement community-based policy promotion to improve immunization uptake.

Notable differences were observed across cities in both vaccine awareness and uptake among older adults. Cities such as Beijing and Hangzhou, characterized by higher socioeconomic development, denser healthcare resources, and more active health education programs, generally demonstrated greater awareness of influenza vaccination among older adults. In contrast, cities like Chongqing and Qingdao showed comparatively lower awareness, which may reflect differences in local health communication, public engagement, and primary healthcare outreach [21]. Differences in vaccination uptake, on the other hand, appear to be influenced not only by awareness but also by local policy environments and financial accessibility. China has implemented heterogeneous reimbursement policies for influenza vaccination across different regions. In most areas, vaccination remains self-paid. Since 2020, Hangzhou has provided free influenza vaccines to adults aged 70 and above. This study demonstrates that Hangzhou achieved significantly higher vaccination coverage compared to other cities, indicating that policy-driven financial accessibility serves as a core driver for vaccine uptake [22]. Although the COVID-19 pandemic heightened public willingness for vaccination, only cost-free provision effectively translates this intention into actual vaccination behavior [23]. Governments or other service organizations should consider subsidized services and vigorously promote free influenza vaccination policies [10]. Interestingly, despite the substantial difference in vaccine awareness between Beijing and Chongqing, their vaccination rates were relatively similar. This pattern implies that awareness alone does not necessarily lead to action; other contextual factor may play mediating or compensatory roles.

Research reveals that in China, several key factors significantly influenza vaccination decisions among the elderly population, including educational background, income levels, marital status, and health insurance coverage. Individuals with health insurance demonstrate higher vaccination rates, a pattern that aligns with observations from North American research [24]. Substantial evidence confirms the role of socio-demographic factors [21]: those cohabiting with spouses or family members exhibited higher vaccination rates [25,26], as social networks and family recommendations positively influence health information acquisition [27]. As people aged, they were more likely to get vaccinated, while higher income [25] and elevated educational attainment [25,28,29] or socioeconomic status [30,31] were identified as potential facilitators of vaccination among the elderly. Notably, we found no significant association between gender and vaccination rates, whereas international evidence remains mixed: some research indicates higher coverage among men [32,33,34,35,36], other studies suggest women are more likely to be vaccinated [24,37,38,39], and some report no significant gender differences [40].

Older adults with poorer health profiles—particularly those with limited self-care capabilities, multiple chronic conditions, hyperlipidemia, and respiratory issues—demonstrated higher vaccination rates. These results are consistent with studies from Europe [41] and Singapore [42], where more chronic diseases correlated with higher vaccination rates. Multiple studies confirm that health conditions, especially specific chronic diseases [39,43,44,45,46,47], influence vaccination decisions among the elderly. A systematic review also discovered that globally, individuals with chronic diseases receive influenza vaccinations at higher rates than the general population [20]. Given their increased susceptibility to influenza and elevated risk of severe complications—including disease exacerbation, pneumonia, multi-organ failure, and mortality—vaccination is particularly critical for this demographic. COPD stood out as a key factor, likely because these patients have weaker cellular immune responses to flu and depend more on vaccine-protected antibody immunity. To improve vaccination coverage, focused strategies should prioritize high-risk populations, including those with chronic conditions such as hyperlipidemia or COPD, alongside socioeconomically vulnerable groups. As this study draws on representative samples from six major Chinese cities, its findings demonstrate robust credibility and reliability, providing valuable supplementary evidence to existing research.

Among vaccinated participants, four main motivation types were identified: family recommendation, comprehensive confidence, clinician-guided, and self-reliant confidence. Family recommendation participants relied on family advice rather than professional guidance; comprehensive confidence participants showed high motivation across multiple factors; clinician-guided participants were primarily influenced by physicians; and self-reliant confidence participants trusted vaccine safety independently. Among unvaccinated participants, four barrier types emerged: Safety Concern, Low Risk Perception, social influence, and perceived vaccine ineffectiveness. Safety concerns, low perceived risk, social influence, and doubts about efficacy were the main reasons for non-vaccination. Overall, vaccinated individuals were driven by external guidance, while unvaccinated individuals were influenced mainly by safety, risk perception, and social factors. These findings highlight the need to tailor vaccination strategies to the specific motivations and barriers of different groups.

Failure to recognize the increased susceptibility to influenza resulting from the combined effects of age and medical risk factors is a primary reason for reduced vaccination willingness. A reluctance to get vaccinated was also linked to inadequate knowledge of the vaccine’s benefits and safety concerns. Such health beliefs have indeed been identified in international studies as prominently affecting influenza vaccination behaviors among the general older population [48,49]. The study also revealed that recommendations from healthcare providers and influence from family and friends are key factors promoting vaccination behavior. Being part of a social circle was found to boost the likelihood of getting vaccinated [50]. Therefore, the next critical step to improving vaccination rates lies in leveraging the endorsements and recommendations of medical professionals to address the two major barriers—misconceptions about influenza and concerns about vaccine safety—while also improving knowledge and attitudes. Emphasis should be placed on the supportive role of family and informal social networks. Primary healthcare institutions should not only ensure service accessibility but also allocate sufficient consultation time to thoroughly discuss the benefits and risks of vaccine protection with patients. Furthermore, regular health education initiatives on influenza and its vaccination should be carried out to enhance awareness, attitudes, and practices among older adults.

Consistent with previous research findings [51,52], our study shows that past influenza vaccination experiences greatly enhance subsequent vaccination behavior among older adults in China. A notable cognitive difference was also observed between vaccinated and unvaccinated groups: over 70% of vaccinated individuals affirmed the vaccine’s effectiveness and expressed willingness to be revaccinated the following year, whereas nearly 90% of unvaccinated older adults reported no intention to vaccinate in the coming year. These results suggest that health administrative departments should seize critical windows for intervention. Encouraging currently unvaccinated individuals to receive the vaccine may initiate a “carry-over effect”, fostering ongoing vaccination in subsequent seasons.

Regular medical check-ups received by older adults present a valuable opportunity for promoting vaccination. Organized immunization counseling in primary healthcare settings can provide evidence-based information to correct misconceptions, biases, and negative attitudes toward vaccination. Counseling should include factual information, such as that influenza vaccines are not 100% effective [53] and may have side effects [54]. Emphasis should be placed on educating high-risk groups about how influenza can exacerbate their underlying conditions, while annual vaccination can effectively reduce infection and prevent severe complications. Simple interventions such as personalized postcards or telephone reminders [55], as well as recommendations from healthcare professionals [56,57,58,59,60,61], can positively influence vaccination rates. Practice has demonstrated that home-visit vaccination services can also effectively improve coverage. Godoy et al. [62] specifically highlighted that patients whose physicians had been vaccinated showed significantly higher vaccination rates than those whose physicians had not. At the systems level, policy interventions may further enhance vaccination rates. Population-based influenza vaccination strategies can also leverage information systems to enable efficient tracking, vaccination, and monitoring of high-risk groups.

This study has several limitations. First, participants were recruited from CHSCs, which may introduce potential selection bias, as older adults who attend CHSCs for health consultations may have higher health awareness and better access to medical services. Second, because the study covered six major urban cities, the findings may not be generalizable to rural or semi-urban populations where healthcare accessibility and vaccination policies differ. Third, the cross-sectional design precludes causal inference. Future studies using longitudinal designs and nationally representative samples are warranted to provide more comprehensive evidence for nationwide policy formulation.

## 5. Conclusions

Our findings indicate that influenza vaccination coverage among older adults in China remains below the World Health Organization’s target. Higher uptake was observed in socioeconomically advantaged groups with better access to healthcare, often despite poorer functional health. Targeted interventions are needed for unmarried, less educated, and uninsured older adults. Major barriers include misconceptions about influenza, social influence from unvaccinated peers or family, and concerns regarding vaccine safety and effectiveness. Strategies to improve coverage should leverage primary healthcare services, structured immunization counseling, family and social networks, personalized reminders, and policy-supported financial incentives.

## Figures and Tables

**Table 1:** Influenza vaccine awareness by socio-demographic and health-related characteristics among urban community-dwelling Chinese adults aged ≥ 60 years (n = 13,363).

Variable		N (%)	Aware (%)	*p*-Value *
Socio-demographic characteristic	Region			0.015
	Beijing	2180 (16.3)	2027 (93.0)	
	Chengdu	2059 (15.4)	1527 (74.2)	
	Chongqing	2523 (18.9)	1665 (66.0)	
	Hangzhou	2208 (16.5)	1959 (88.7)	
	Qingdao	2140 (16.0)	1567 (73.2)	
	Shenzhen	2253 (16.9)	1756 (77.9)	
	Gender			1.000
	Male	6048 (45.3)	4712 (77.9)	
	Female	7315 (54.7)	5789 (79.1)	
	Age (year)			0.015
	60–65	3825 (28.7)	3063 (80.1)	
	65–70	3843 (28.8)	3052 (79.4)	
	70–75	2755 (20.6)	2141 (77.7)	
	75–80	1547 (11.6)	1206 (78.0)	
	80–85	785 (5.9)	582 (74.1)	
	≥85	598 (4.5)	457 (76.4)	
	Education			0.015
	Low	3720 (27.8)	2429 (65.3)	
	Medium-low	3992 (29.8)	3125 (78.3)	
	Medium-high	3461 (25.9)	2959 (85.5)	
	High	2190 (16.4)	1978 (90.3)	
	Monthly Personal Income			0.015
	≤2500 CNY	3850 (28.8)	2440 (63.4)	
	2501–5000 CNY	5829 (43.6)	4836 (83.0)	
	5001–7500 CNY	2457 (18.4)	2145 (87.3)	
	≥7500 CNY	1227 (9.2)	1080 (88.0)	
	Marital Status			0.015
	Married/Cohabiting	11,658 (87.2)	9284 (79.6)	
	Single/widowed	1705 (12.8)	1217 (71.4)	
	Urban Employee Basic Medical Insurance			0.015
	Yes	7919 (59.2)	6779 (85.6)	
	No	5444 (40.8)	3722 (68.4)	
	Urban-Rural Resident Basic Medical Insurance			0.015
	Yes	4840 (36.2)	3391 (70.1)	
	No	8523 (63.8)	7110 (83.4)	
Health-related characteristic	Self-care ability			0.015
	Yes	11,319 (84.7)	9061 (80.0)	
	No	2044 (15.3)	1440 (70.5)	
	Self-assessed health status			0.015
	Poor	1403 (10.5)	968 (69.0)	
	Fair	5367 (40.2)	4192 (78.1)	
	Good	6593 (49.3)	5341 (81.0)	
	Hypertension			0.015
	Yes	6234 (46.7)	4993 (80.1)	
	No	7129 (53.3)	5508 (77.3)	
	Diabetes			1.000
	Yes	3186 (23.8)	2518 (79.0)	
	No	10,177 (76.2)	7983 (78.4)	
	Hyperlipidemia			0.015
	Yes	2183 (16.3)	1840 (84.3)	
	No	11,180 (83.7)	8661 (77.5)	
	Stroke/Cerebrovascular Disease			0.885
	Yes	517 (3.9)	389 (75.2)	
	No	12,846 (96.1)	10,112 (78.7)	
	COPD/Respiratory diseases			1.000
	Yes	676 (9.1)	526 (77.8)	
	No	12,687 (94.9)	9975 (78.6)	

* *p*-values were adjusted for multiple χ^2^ tests using the Bonferroni correction (m = 15). Adjusted *p* < 0.05 was considered statistically significant.

**Table 2:** Influenza vaccination rates by socio-demographic and health-related characteristics among urban community-dwelling Chinese adults aged ≥ 60 years (n = 13,363).

Variable	Vaccinated N (%)	*p*-Value *
Awareness		0.018
Yes	4233 (40.3)	
No	317 (11.1)	
Region		0.018
Beijing	708 (32.5)	
Chengdu	645 (31.3)	
Chongqing	801 (31.7)	
Hangzhou	1013 (45.9)	
Qingdao	600 (28.0)	
Shenzhen	783 (34.8)	
Gender		1.000
Male	2110 (34.9)	
Female	2440 (33.4)	
Age (year)		1.000
60–65	1313 (34.2)	
65–70	1327 (34.5)	
70–75	911 (33.1)	
75–80	545 (35.2)	
80–85	251 (32.0)	
≥85	44 (42.3)	
Education		0.018
Low	1043 (28.0)	
Medium-low	1383 (34.6)	
Medium-high	1256 (36.3)	
High	868 (39.6)	
Monthly Personal Income		0.018
≤2500 CNY	1001 (26.0)	
2501–5000 CNY	2153 (36.9)	
5001–7500 CNY	937 (38.1)	
≥7500 CNY	459 (37.4)	
Marital Status		0.018
Married/Cohabiting	4040 (34.7)	
Single/widowed	510 (29.9)	
Urban Employee Basic Medical Insurance		0.018
Yes	2850 (36.0)	
No	1700 (31.2)	
Urban-Rural Resident Basic Medical Insurance		0.018
Yes	1560 (35.1)	
No	2990 (32.2)	
Self-care ability		0.594
Yes	3812 (33.7)	
No	738 (36.1)	
Self-assessed health status		0.558
Poor	465 (33.1)	
Fair	1768 (32.9)	
Good	2317 (35.1)	
Number of comorbidities		0.126
zero	1470 (33.0)	
One	1721 (33.7)	
Two	833 (34.7)	
Three or more	526 (37.9)	
Hypertension		1.000
Yes	2169 (33.8)	
No	2381 (33.4)	
Diabetes		1.000
Yes	1128 (35.4)	
No	3422 (33.6)	
Hyperlipidemia		0.198
Yes	795 (36.4)	
No	3755 (33.6)	
Stroke/Cerebrovascular Disease		1.000
Yes	517 (31.1)	
No	12846 (34.2)	
COPD/Respiratory diseases		0.126
Yes	263 (38.9)	
No	4287 (33.8)	
Self-reported Indicative of flu immunization		0.018
Eligible	3377 (74.2)	
Ineligible	1173 (25.8)	

* *p*-values were adjusted for multiple χ^2^ tests using the Bonferroni correction (m = 15). Adjusted *p* < 0.05 was considered statistically significant.

**Table 3:** Socio-demographic factors affecting influenza vaccine uptake in the previous 12 months among urban community-dwelling Chinese adults aged ≥60 years (n = 13,363).

Variables	Count	Univariate Logistic Regression			Multivariate Logistic Regression		
		OR	95% CI	*p*-Value	OR	95% CI	*p*-Value
Gender							
Female	7315	1	1		1	1	
Male	6048	1.071	0.996–1.150	0.063	1.029	0.956–1.107	0.445
Age							
60–<65	3835	1	1		1	1	
65–<70	3843	1.013	0.922–1.113	0.787	1.107	0.925–1.118	0.729
60–<75	2755	0.949	0.855–1.053	0.322	0.982	0.884–1.091	0.737
75–<80	1547	1.045	0.923–1.182	0.388	1.123	0.990–1.275	0.071
80–<85	785	0.903	0.766–1.064	0.222	0.990	0.837–1.171	0.908
≥85	104	1.409	0.949–2.090	0.09	1.114	0.920–1.348	0.269
Education							
Low	3720	1	1		1	1	
Medium-low	3992	1.361	1.235–1.499	<0.001	1.181	1.062–1.313	0.002
Medium-high	3461	1.462	1.321–1.615	<0.001	1.193	1.060–1.344	0.004
High	2190	1.462	1.507–1.884	<0.001	1.382	1.196–1.597	<0.001
Monthly Personal Income							
≤2500 CNY	3850	1	1		1	1	
2501–5000 CNY	5825	1.667	1.524–1.823	<0.001	1.589	1.432–1.764	<0.001
5001–7500 CNY	2457	1.755	1.574–1.956	<0.001	1.583	1.383–1.813	<0.001
7500 CNY	1227	1.701	1.484–1.949	<0.001	1.468	1.236–1.743	<0.001
Marital Status							
Single/widowed	1705	1	1		1	1	
Married/Cohabiting	11,658	1.243	1.113–1.388	<0.001	1.153	1.026–1.296	0.016
Urban Employee Basic Medical Insurance							
No	5446	1	1		1	1	
Yes	7917	1.239	1.152–1.334	<0.001	1.124	0.983–1.287	0.088
Urban-Rural Resident Basic Medical Insurance							
No	8523	1	1		1	1	
Yes	4840	1.136	1.054–1.225	<0.001	1.270	1.111–1.452	<0.001

**Table 4:** Health status factors affecting influenza vaccine uptake in the previous 12 months among urban community-dwelling Chinese adults aged ≥ 60 years (n = 13,363).

Risk Factor	Count	Univariate Logistic Regression			Multivariate Logistic Regression		
		OR	95% CI	*p*-Value	OR	95% CI	*p*-Value
Self-care ability							
Yes	11,319	1	1		1	1	
No	2044	1.113	1.009–1.228	0.033	1.154	1.041–1.280	0.006
Self-assessed health status							
Poor	1403	1	1		1	1	
Average	5367	0.991	0.875–1.123	0.887	1.044	0.917–1.187	0.517
Good	6593	1.093	0.967–1.235	0.153	1.218	0.067–1.391	0.003
Hypertension							
No	7129	1	1	0.090	1	1	
Yes	6234	1.064	0.990–1.143		1.068	0.991–1.152	0.084
Diabetes							
No	10,177	1	1		1	1	
Yes	3186	1.082	0.995–1.176	0.064	1.083	0.993–1.182	0.073
Hyperlipidemia							
No	11,180	1	1		1	1	
Yes	2183	1.133	1.029–1.246	0.010	1.133	1.026–1.251	0.014
Stroke/Cerebrovascular Disease							
No	12,846	1	1		1	1	
Yes	517	0.871	0.721–1.053	0.155	0.848	0.697–1.031	0.098
COPD/Respiratory diseases							
No	12,687	1	1		1	1	
Yes	676	1.248	1.064- 1.463	0.006	1.294	1.101–1.521	0.002

**Table 5:** Network analysis of co-occurring reasons for vaccination among vaccinated participants.

Reason	Degree	Betweenness	Closeness	Eigenvector
Safe/effective	7	0	8.278	0.954
Family recommendation	7	0	7.610	1.000
Doctor recommendation	7	0	8.756	0.820
Social media	7	14	0.002	0.283
Prior helpful	7	0	0.001	0.687
Government recommendation	7	0	9.930	0.530
Protect family	7	0	0.001	0.572
Affordable	7	0	0.001	0.330

**Table 6:** Network analysis of co-occurring reasons for non-vaccination among unvaccinated participants.

Reason	Degree	Betweenness	Closeness	Eigenvector
Mild influenza symptoms	8	0	0.001	0.999
Perceived vaccine ineffectiveness	8	0	0.001	0.877
Family and friends influence	8	0	0.001	0.883
Doctor recommendation	8	26	0.003	0.215
Social media influence	8	0	0.002	0.551
Concerns about side effects	8	0	0.001	0.941
Commercial motives	8	0	0.002	0.458
Access inconvenience	8	0	0.002	0.473

## Data Availability

The data within this article will be shared upon reasonable request to the corresponding author.

## Supplementary Materials

The following supporting information can be downloaded at: https://www.mdpi.com/article/10.3390/vaccines13111171/s1, File S1: Questionnaire Structure and Content; File S2: Attitude scores toward influenza vaccination among vaccinated and unvaccinated participants; File S3: Proportions of vaccination reasons endorsed within each cluster among vaccinated participants; File S4: Proportions of non-vaccination reasons endorsed within each cluster among unvaccinated participants.
