# Supplementary material for: Factors Associated with Influenza Vaccination Among Urban Community-Dwelling Chinese Elderly: Results from a Multicity Cross-Sectional Study
Source: Vaccines (Basel). 2025 Nov 18;13(11):1171. doi: 10.3390/vaccines13111171 (PMC12656644; doi:10.3390/vaccines13111171)
Supplement: Supplementary file 1 [file vaccines-13-01171-s001.zip › vaccines-3946682-supplementary.pdf]

File S1: Questionnaire Structure and Content

Hello. This questionnaire is organized and implemented by the *Peking Union Medical College, Chinese Academy of Medical Sciences*, aiming to understand the vaccination behavior and intention of the elderly, and we hope that you can answer truthfully according to your own situation, and we thank you for your understanding and support. Your answers and participation will help us to better develop and optimize vaccine education programs. All information collected about you in the questionnaire will be used for the research of this evaluation survey and will not be disclosed to a third party or used for purposes other than this evaluation without your consent.

If you are willing to participate in this survey, please tick and sign your name.

I agree to participate in the survey □

Please sign your name: _________

Section	Q#	Question	Options
1. Socioeconomic and Demographic Variables	1	Gender	(1) Male (2) Female
	2	Date of birth	Year ___ Month ___
	3	Marital status	(1) Single (2) Married (3) Divorced (4) Widowed
	4	Education level	(1) Primary school or below (2) Junior high school (3) High school / Vocational / Technical school (4) College / Bachelor's degree (5) Master's degree or above
	5	Type of health insurance (multiple choice allowed)	(1) Urban Employee Basic Medical Insurance (2) Urban Resident Basic Medical Insurance
	6	Monthly income (including all sources)	(1) < 2,000 CNY (2) 2,000–5,000 CNY (3) 5,001–10,000 CNY (4) > 10,000 CNY
2. Health Status Variables	7	Primary caregiver	(1) Spouse (2) Children (3) Nanny / Care worker (4) Self
	8	Have you ever been diagnosed by a doctor with any of the following conditions? (multiple choice allowed)	(1) Hypertension (2) Diabetes (3) Hyperlipidemia (4) Cardiovascular diseases (e.g., coronary heart disease, angina, myocardial infarction) (5) Cerebrovascular diseases (e.g., stroke, cerebral infarction, cerebral hemorrhage) (6) Respiratory diseases (e.g., asthma, COPD, chronic

			bronchitis) (7) Tumors / Cancer (8) Other
	9	How would you rate your health status over the past year?	(1) Very poor (2) Poor (3) Fair (4) Good (5) Very good
	10	Are you eligible for the influenza vaccine?	(1) Yes (2) No
3. Awareness and Attitudes toward Influenza Vaccination	11	Have you heard of the influenza vaccine?	(1) Yes (2) No
	12	Have you received an influenza vaccine in the past 12 months?	(1) Yes → go to 11.1 (2) No → go to 11.2
	12.1.1	Do you think receiving the influenza vaccine benefits you? (for vaccinated respondents)	(1) Very beneficial (2) Quite beneficial (3) Neutral (4) Not very beneficial (5) Not beneficial at all
	12.2.1	Do you think your health condition requires influenza vaccination to prevent the flu? (for unvaccinated respondents)	(1) Very necessary (2) Quite necessary (3) Uncertain (4) Not very necessary (5) Not necessary at all
4. Reasons for Accepting Vaccination	12.1.2	Which of the following reasons contributed to your decision to get vaccinated? (Multiple choice allowed) (for vaccinated respondents)	1. Believe vaccine is safe and effective 2. Family/friends recommended 3. Doctor recommended 4. Learned benefits from social media 5. Previously vaccinated and found helpful 6. Government recommendation 7. Protect family 8. Reasonable/free price
	12.1.2.1	Among the reasons you selected, choose the one that influenced you the most	(1) [one selected reason] (2) [one selected reason] (3) [one selected reason]
4. Reasons for Declining Vaccination	12.2.2	Which of the following reasons contributed to your	1. Never heard of vaccine 2. Believe vaccination cannot prevent flu 3. Family/friends not vaccinated 4. Doctor

		decision not to get vaccinated? (Multiple choice allowed) (for unvaccinated respondents)	assessment 5. Negative reports online 6. Concern about side effects 7. Believe promotion driven by commercial interests 8. Prefer natural immunity 9. Inconvenient time/location 10. Don't know when/where to vaccinate 11. Too expensive
	12.2.2.1	Among the reasons you selected, choose the one that influenced you the most	(1) [one selected reason] (2) [one selected reason] (3) [one selected reason]
5. Intention for Revaccination	12.1.3	Do you plan to continue receiving the influenza vaccine in the coming year? (for vaccinated respondents)	(1) Yes, continue vaccination (2) No, not continue vaccination
	12.2.3	Do you plan to get the influenza vaccine in the coming year? (for unvaccinated respondents)	(1) Yes (2) No (3) Not sure / Undecided

File S2: Comparison of attitude scores toward influenza vaccination between vaccinated and unvaccinated participants (n=13363)

Vaccination Status	n	Attitude Score (Mean)	t	P
Unvaccinated	8,813	2.61	-87.252	<0.001
Vaccinated	4,550	4.03		

File S3: Proportions of vaccination reasons endorsed within each cluster among vaccinated participants

cluster	Safe/effective	Family recommendation	Doctor recommendation	Social media	Prior helpful	Government recommendation	Protect family	Affordable
1	0.151432469	0.607094134	0.122783083	0.082764893	0.223283311	0.132332879	0.141882674	0.065029559

2	1	0.717557252	1	0.162849873	0.312977099	0.239185751	0.20610687	0.122137405
3	0	0	1	0.034632035	0.056277056	0.103896104	0.047619048	0.03030303
4	1	0	0	0.014037433	0.10026738	0.059491979	0.046122995	0.022727273

File S4: Proportions of non-vaccination reasons endorsed within each cluster among unvaccinated participants

cluster	Mild influenza symptoms	Perceived vaccine ineffectiveness	Family and friends influence	Doctor recommendation	Social media influence	Concerns about side effects	Commercial motives	Access inconvenience	High vaccination cost
1	0.108962554	0.084407612	0.088704727	0.14640884	0.142725599	0.503990178	0.121240025	0.171884592	0.188766114
2	1	0.093446221	0.085293195	0.009720916	0.01505174	0	0.014738162	0.021323299	0.03480715
3	0	0.06916996	1	0.028985507	0.038208169	0	0.027667984	0.053359684	0.030961792
4	0	1	0	0.02004717	0.02240566	0	0.04009434	0.027122642	0.04009434
